# Hydrogels in Soft Robotics: Past, Present, and Future

**DOI:** 10.1021/acsnano.3c12200

**Published:** 2024-08-05

**Authors:** Antonio López-Díaz, Andrés S. Vázquez, Ester Vázquez

**Affiliations:** † Escuela Técnica Superior de Ingeniería Industrial, Universidad de Castilla−La Mancha, 13071, Ciudad Real, Spain; ‡ Instituto Regional de Investigación Científica Aplicada, Universidad de Castilla−La Mancha, 13071, Ciudad Real, Spain; § Facultad de Ciencias y Tecnologías Químicas, Universidad de Castilla−La Mancha, 13071, Ciudad Real, Spain

**Keywords:** Hydrogels, soft robotics, soft actuators, soft sensors

## Abstract

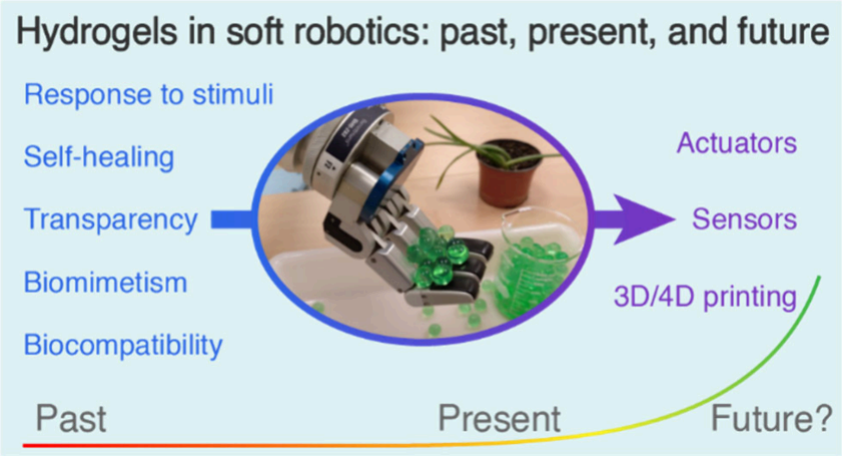

The rise of soft robotics in recent years has motivated significant developments in smart materials (and vice versa), as these materials allow for more compact robotic designs thanks to the embodied intelligence that they provide. Hydrogels have long been postulated as one of the potential candidates to be used in soft robotics due to their softness, elasticity, and smart properties that can be tuned with nanomaterials. However, nowadays they represent only a small percentage of the materials used in the field. In this perspective, the drawbacks that have hindered their utilization so far are analyzed as well as the current state of hydrogel-based soft actuators, sensors, and manufacturing possibilities. The future improvements that need to be made to achieve a real application of hydrogels in soft robotics are also discussed.

The field of *soft robotics* has experienced a huge growth in the past decade. Unlike in traditional robotics, where engineering disciplines such as mechanics, electronics, or software cover the majority of research, materials play a key role in soft robots. In this regard, typical elastomers such as rubbers and silicones have been extensively used in the field due to their elasticity and compliance. Nonetheless, the current trends are shifting toward *smart soft materials*, as they allow the development of more compact and capable robots, with *embodied intelligence*, without the need to complicate designs or control strategies^1^ (for example, the same material can be used as structure and sensor all-in-one). Ultimately, with smart materials, the final aim is to replicate the capabilities of living beings in robots, which gives rise to the term *bioinspiration*.

*Hydrogels* are the materials that most closely resemble biological tissues^2,3^ and, more importantly, they can exhibit different smart properties that can be tuned thanks to the addition of nanomaterials,^4^ so *a priori* they are postulated as ideal candidates for soft robotics. However, their use in the field has never been as significant as expected, although a positive trend is observed in the past few years, also motivated by the growth of soft robotics.

In what follows, we attempt to summarize the career path of hydrogels in soft robotics, the drawbacks that have hindered their use in the field, the current state, and the challenges that need to be solved to achieve a meaningful contribution, highlighting the role of nanomaterials.

## Hydrogel-Based Soft Actuators

**Figure 1:**
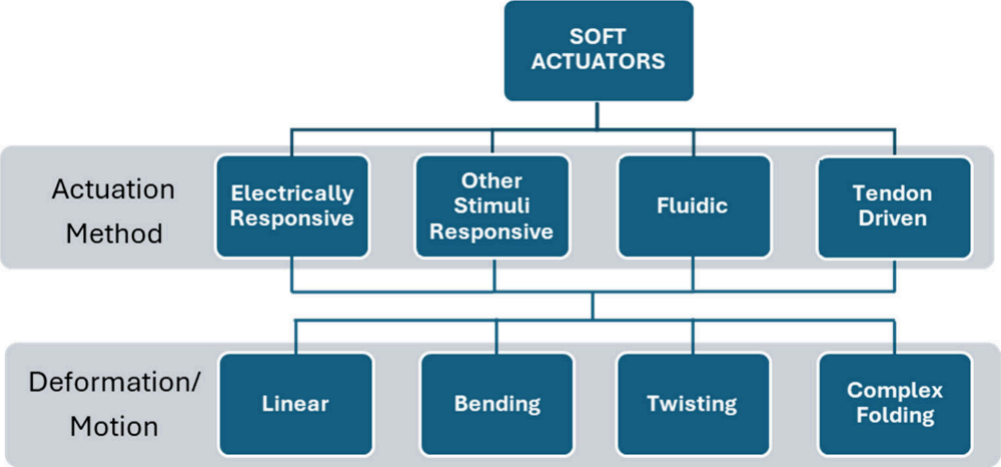
Classification of soft actuators is dependent on the actuation method and the motion principle. Theoretically, any actuation method could yield any movement, although certain combinations would be more challenging. For instance, fluidic actuation may face difficulty in achieving complex folding due to the limited geometry of fluidic actuators.

Hydrogel-based soft actuators allow soft robots to perform various functions or actions mainly by controlling the deformation of morphing materials.^5^ For example, hydrogel-based actuators can allow soft robots to grasp delicate objects.^6^ They also enable soft robots to perform locomotive actions such as swimming,^7^ walking and crawling^8^ or even jumping.^9^ To understand the utilization of hydrogels in these soft actuators, it is necessary to first present an overview of the different types of soft actuation. The motion exhibited by soft actuators arises from the deformations in their shapes induced by an actuation method. This method can take advantage of the material’s response to stimuli (such as electric or magnetic fields, light, or temperature) or is based on fluidic or tendon-driven mechanisms. The deformations result in motions such as expansion/contraction, bending, twisting, or more complex folding. Figure 1 summarizes a classification based on the nature of the motion and actuation method. Through this classification, we present the various applications of hydrogels in soft actuators that have emerged over time.

### Electrically Responsive Actuators

Soft electric actuators rely on smart electroactive polymers (EAP). Their shape deformation is mainly driven by the mobility of ions, as seen in ionic polymer–metal composites (IPMC), or by electrostatic forces, as observed in Dielectric Elastomers (DE). Ionic hydrogels have also been researched for electronic actuation, mainly for bending motion, for a long time. The initial studies focused on actuation in aqueous media, while recently, research on *out of water* has been conducted.

#### - Underwater Actuation

**Figure 2:**
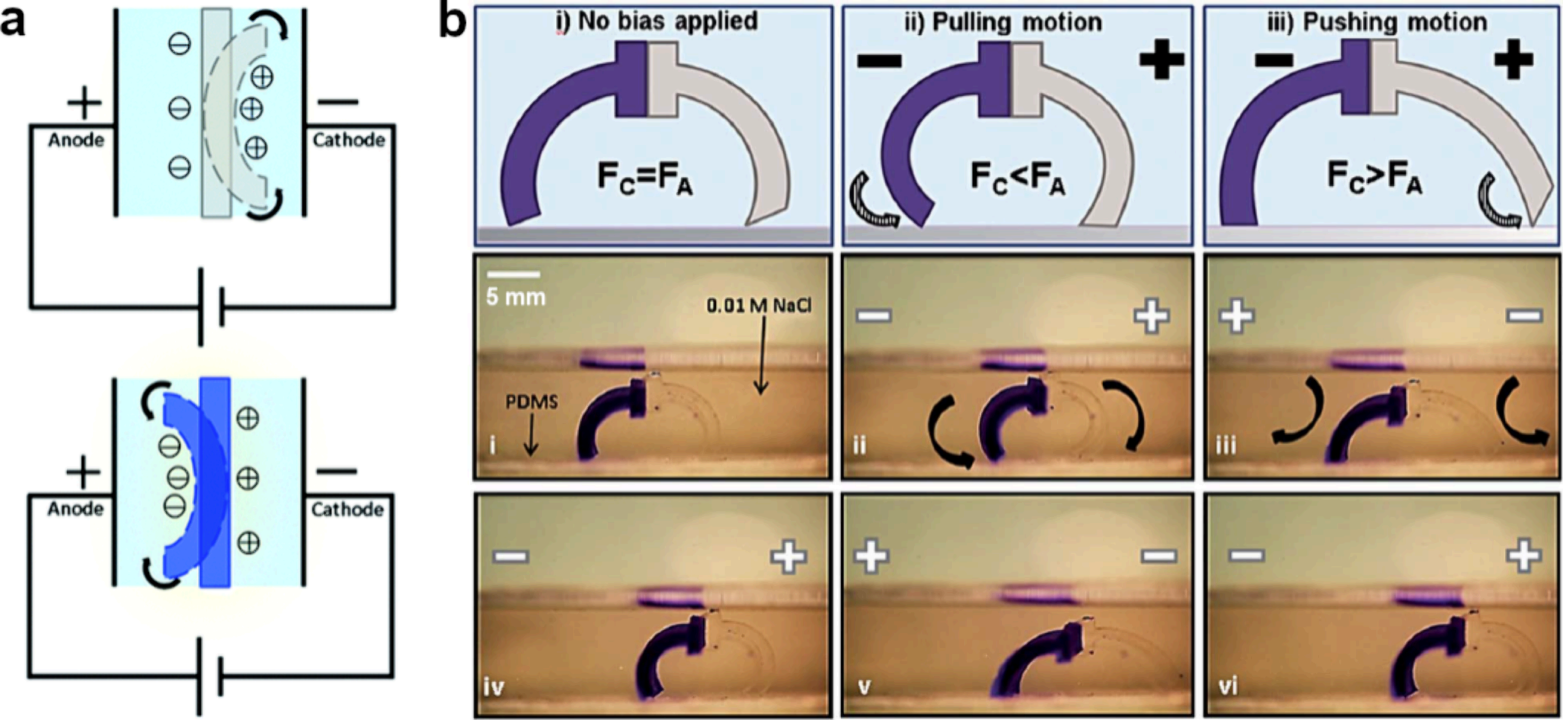
(a) Bending behavior of ionic hydrogels in aqueous solutions under electric fields. The movement of ions results in swelling of the hydrogel on the side near the cathode (cationic network in blue) or near the anode (anionic network in white), causing subsequent bending toward the opposite side. Reprinted with permission from ref (12). Copyright 2014 Royal Society of Chemistry. (b) An anionic hydrogel (white) and a cationic hydrogel (blue) are paired to build a walker robot able to move inside water when the electric field. Reprinted with permission from ref (12). Copyright 2014 Royal Society of Chemistry.

The research journey into hydrogels as actuators began with early investigations of their actuation capabilities in water environments. In 1990, the work of Shiga and Kurauchi demonstrated that ionic hydrogels bend in ionic aqueous solutions under electric fields,^10^ while nonionic networks do not exhibit this behavior. This bending response is caused by changes in the osmotic pressure, originating from ionic motion, which results in different ion concentrations inside and outside of the gel, along with conformational changes in the polymeric network. For instance, in the case of a cationic network, the ionic movement leads to a swelling of the hydrogel on the side close to the cathode and the consequent bending movement toward the anode (Figure 2a).

After this pioneering work, many others tried to exploit this bending motion as the actuation mode for underwater robots.^11^ However, despite the promising impactful beginning of this kind of actuation, it was progressively losing importance due to the lack of immediate application. In the first decade of the 21st century, the impact of studies about this topic was considerably lower than in the 90s. Nevertheless, works about electroactive hydrogels, using the same working principle, have emerged in the past few years motivated by the rise of soft robotics. For underwater locomotion, one example is the walker robot shown in Figure 2b, which is made up of two hydrogels with different electric nature,^12^ or for underwater robotic manipulation.^13^

One of the issues with these actuators is that the electroactive response is quite slow. For example, the largest propulsion velocity achieved by the walker robot (Figure 2b) was ∼2.5 mm min^–1^, equal to moving half the length of his body in 1 min. The thickness is a critical parameter in the speed of the movement. Normally, a lower thickness results in higher speed, but also compromises the strength of the system, so a trade-off must be reached. This feature, intrinsic to the system, can be exploited with creative solutions based on designs with different dimensions to achieve devices that satisfy the requirements of speed/strength of robotic applications. On the other hand, nanomaterials have been frequently used to reduce this response time. In particular, reduced graphene oxide has been proven to speed up and widen the bending response of certain hydrogels.^14^

Another issue is the control of the swelling of these materials. In aqueous conditions, hydrogels with a large number of hydrophilic functional groups and large pores could swell to such an extent that their mechanical properties or conductivity would deteriorate, and consequently, they would lose their performance. However, this problem can easily be tackled from a chemical point of view by using a mixture of hydrophobic and hydrophilic starting monomers, by increasing the cross-linking density or by preparing interpenetrated double networks.^15^

In summary, the electrical actuation of hydrogels and hybrid hydrogels in aqueous media has been studied quite extensively, leading to underwater robotic applications such as those mentioned. However, most robotic applications are needed out of water, as this is the environment in which we live, so to extend the degree of utility of these materials, it is necessary to eliminate the dependence on an external aqueous medium for their operation.

#### - Out of Water Actuation

**Figure 3:**
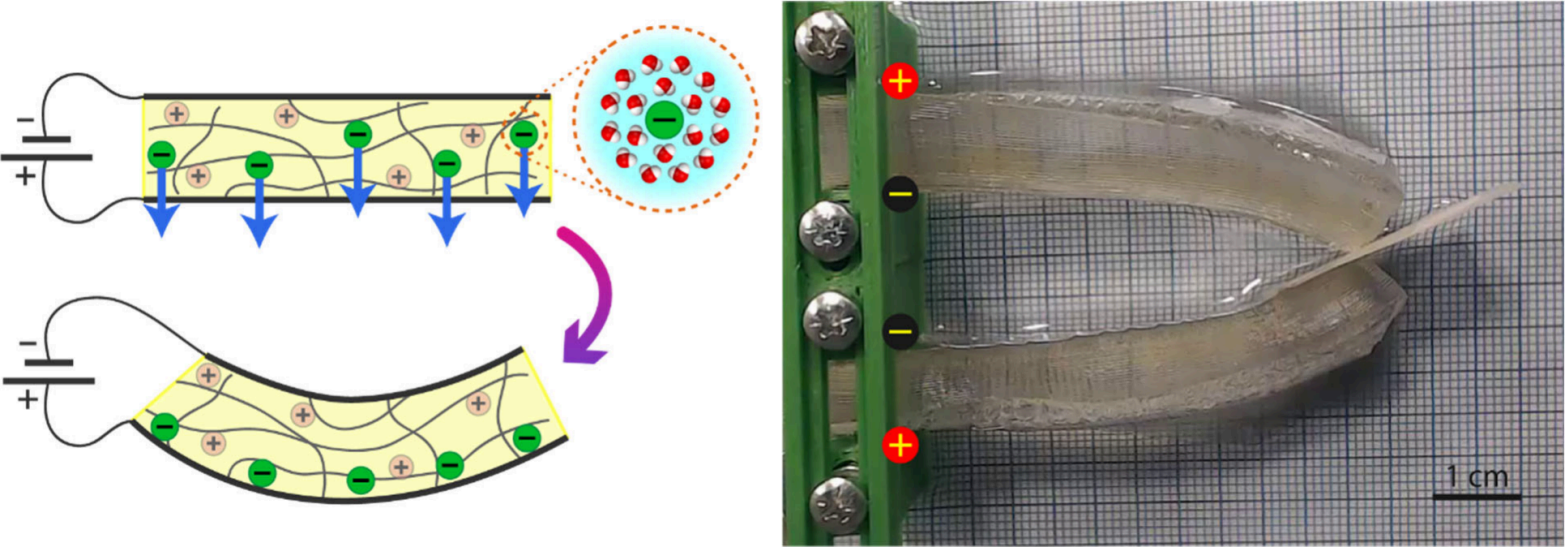
Bending response of our cationic hydrogel under air conditions. The free anions (chlorides) drag the free water contained inside the hydrogel toward the positive electrode, originating a gradient of swelling that bends the hydrogel bar toward the negative electrode. This behavior can be applied to build soft grippers: two parallel hydrogel bars actuated with opposite electric fields (in this case, with the negative pole inside due to the hydrogel’s nature). The electrodes are simple aluminum foil sheets attached to the hydrogel.

A way to generate bending motion with hydrogels is to take advantage of the water contained in the hydrogel. With that in mind, we have recently developed an electroactive hydrogel based on a cationic network that is able to bend outside aqueous media.^16^ Instead of needing a gradient in the osmotic pressure of the external medium, as happened in the cases described before, this hydrogel benefits from its ionic conductivity and its high water absorption capacity to drag the internal water to one side, inducing a swelling on this side and a shrinking on the opposite side, which leads to a bending behavior that can be exploited in robotic grippers (Figure 3). Nevertheless, this approach also poses an issue: how to keep a constant amount of water inside the hydrogel by preventing it from evaporating. To address this challenge, graphene was added to the hydrogel to improve its heat dissipation and, consequently, to reduce the evaporation, playing an important role in the life cycle of this hydrogel-based actuator. Other possible solutions would consist of encapsulating the hydrogel in a thin, elastic, and flexible coating, the creation of hybrid organo-hydrogels, or the introduction of inorganic salts and nanoparticles.^17^ Moreover, as in the underwater actuators, the response is slow. Improved chemical and geometrical designs and the use of nanoparticles would take part in solving this issue for some applications. Despite these drawbacks, in our opinion, the approach of using hydrogels capable of utilizing the water that they contain deserves more attention. It offers the possibility of developing a soft material that responds to electrical currents with a design completely different from that of typical soft actuators.

### Other Stimuli Responsive Actuators

In addition to electricity, other stimuli-responsive methods, such as magnetic fields or light, have been explored. However, these actuation methods are often implemented as proof of concept or small-scale robots. For hydrogel-based actuators, it is of interest to divide them, again, into those that require surrounding water and those that operate in air.

#### - Underwater Actuation

**Figure 4:**
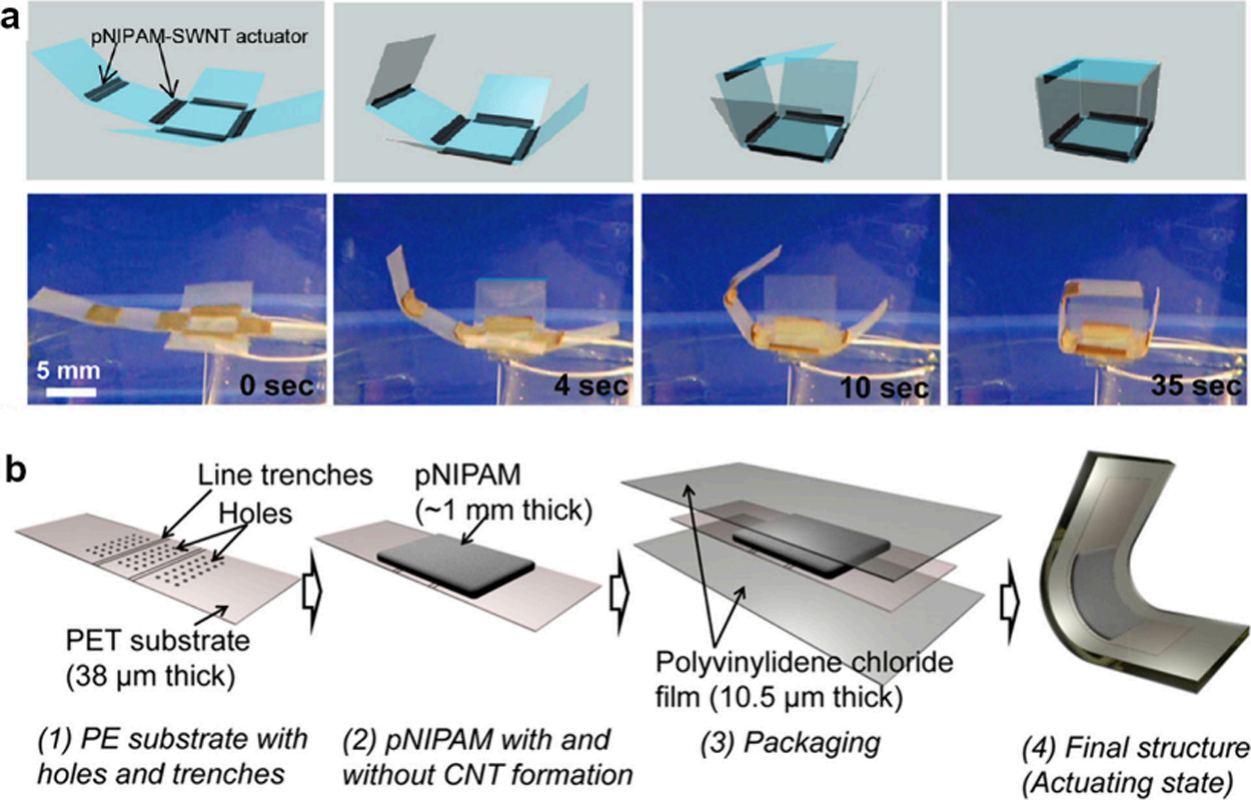
(a) Folding cube, in aqueous media, based on thermally responsive pNIPAM/LDPE bilayer actuators. Reprinted with permission from ref (18). Copyright 2011 American Chemical Society (b) Packaged pNIPAM actuator for working under air conditions. Reprinted with permission from ref (19). Copyright 2015 American Chemical Society

Nanomaterials have played a key role in the development of hydrogel-based underwater actuators that respond to stimuli other than electrical. Take the case of the poly(*N*-isopropylacrylamide) (pNIPAM) hydrogel loaded with single-walled carbon nanotubes developed by Zhang et al.,^18^ which responds to temperature when it is immersed in water and is used to create underwater folding structures (Figure 4a). The added carbon nanotubes not only enhance the thermal response (5 times better compared to pure pNIPAM hydrogel) but also allow a fast optical response thanks to their strong near-infrared absorption (pure pNIPAM hydrogel is transparent in this wavelength).

#### - Out of Water Actuation

Some authors have explored encapsulating the hydrogel with the water necessary for their actuation. That is, allowing the hydrogel to always have a layer of water around it. The thermal-, light-responsive actuator presented by Yamamoto et al. represents an example of this approach:^19^ a pNIPAM hydrogel is packaged together with water in a polyvinylidene chloride thin film (Figure 4b). When the actuator is heated, the hydrogel loses water and shrinks, provoking the actuator bending. But the released water is not lost; it remains in the package, which makes the swelling possible when the hydrogel is cooled, thus enabling the repetitive operation of the actuator. Besides, carbon nanotubes are added to make the response faster, wider, and more stable and also to provide optical response by converting light into heat thanks to their absorption capability. Other authors have explored different approaches for actuation, for example, synthesizing hybrid hydrogels embedded with ferrous nanoparticles (e.g., iron oxide, Fe_3_O_4_) to respond to magnetic fields.^20^ The magnetic attraction exerted over the ferromagnetic nanoparticles caused the hydrogel to move macroscopically. In these hydrogel-based actuators that can operate in air conditions, the actuation is due to smart properties whose origin can reside in the own hydrogel’s nature (network, composition, etc.) or in nanoparticles added to the matrix.

An important handicap of most stimuli-responsive actuators is that, no matter the origin of the smart features, an external element (lamp, magnet, etc.) positioned in the working environment is needed to produce and guide the robot motion. This compromises the compactness and softness of the robot as many of these triggering elements are rigid. Besides, in some cases, the robot operation is compromised as well. For instance, in the case of light-responsive actuators, obstacles can shade the actuator, making its operation difficult. A similar situation can occur with a field generated from outside, as obstacles can distort the field, causing the actuation to change or even disappear.

Another critical problem shared by soft actuators based on stimuli-responsive hydrogels is their mechanical weakness. This property, useful in other fields such as tissue engineering, entails a disadvantage in stimuli-responsive actuators for classical robots: their practical applications are limited as they do not have enough strength to interact with common objects. Tuning the hydrogel stiffness is essential to obtaining a material that is soft but stiff enough to interact with the environment. This mechanical issue can be addressed through the chemical design of the hydrogel or through the addition of nanomaterials, which is a common method to improve the mechanical performance of materials. Going further, the ideal situation would be the active modulation of the hydrogel stiffness by external stimuli.^21^ In this way, the hydrogel could be stiffer or softer depending on the actuation state, as well as possibly having zones with different stiffness, as happens in our hydrogel-based fingertip demonstrator.^22^ The stiffness modulation is a hot topic in the soft robotics field,^23^ and hydrogels are no exception. In any case, despite the efforts to solve this mechanical problem following the different approaches mentioned, it is still hard to find actual robotic applications, not only potential demonstrators, which use stimuli-responsive hydrogels as the actuation power.

### Fluidic Actuators

**Figure 5:**
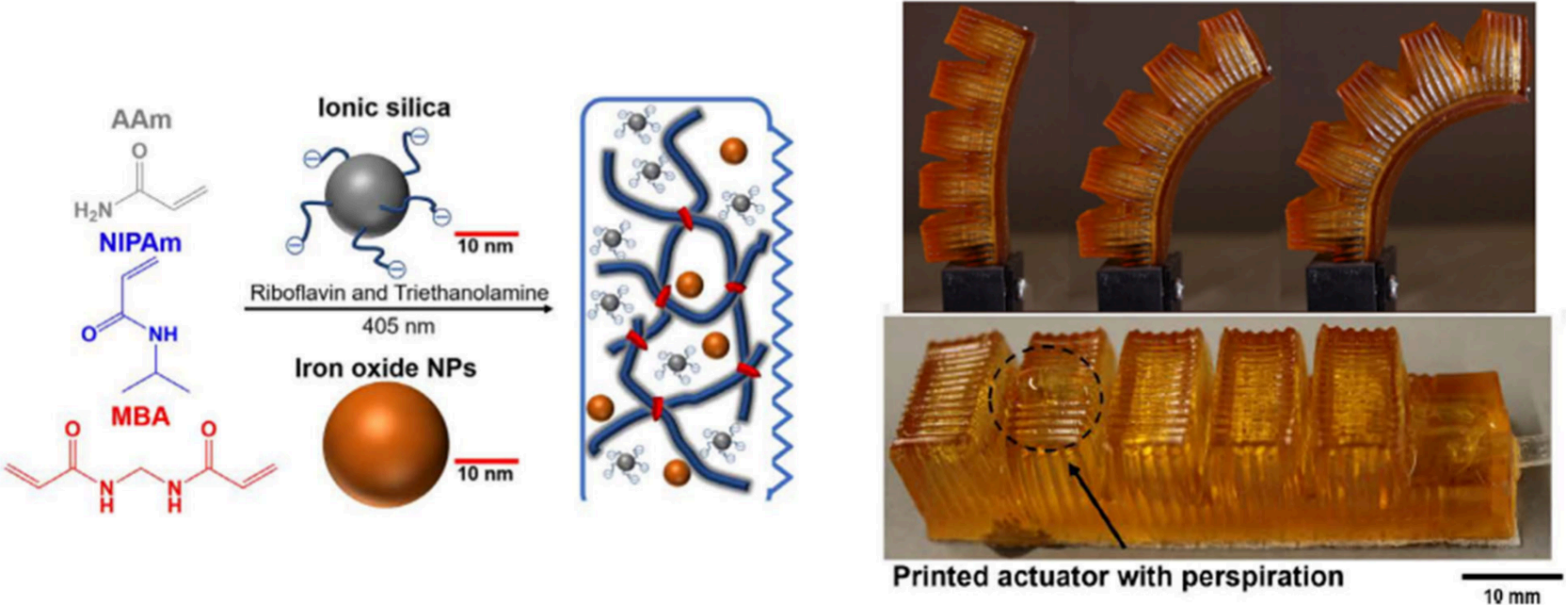
Hybrid pNIPAM and pAAM hydrogels were used to 3D print fluidic bending actuators with perspiration capabilities. Thanks to the chemo-mechanical response of these hydrogels, the pores in the actuator’s structure open and close depending on the temperature, which helps to improve the life cycle of the system. Nanoparticles (iron oxide and silica) are employed to improve the mechanical integrity of the system. Reprinted with permission from ref (24). Copyright 2020 The American Association for the Advancement of Science.

To compensate for the lack of strength, and often speed, that smart hydrogel-based actuators present, other power sources, such as pressurized air, have been recently studied. Fluidic actuators, mainly pneumatic, are widely used in soft grippers and soft manipulators, and they are usually made of inert (i.e., nonsmart) elastomers, since the pressurized fluid is responsible for actuating the system. All fluidic actuators made of hydrogels are operated outside of water to avoid swelling issues. This approach is viable, because water does not act as a motion precursor in this method. The advantages of using hydrogels over other materials to build these actuators lie in the additional capabilities that these materials can confer. For example, Mishra et al. propose a classical pneumatic actuator that benefits from autonomous perspiration due to the chemo-mechanical response of the two hydrogel materials used (pNIPAM and polyacrilamyde, pAAM), which open and close pores in the actuator’s structure depending on the temperature (Figure 5).^24^ Thanks to this capacity, the life cycle of the actuator can be prolonged. Besides, iron oxide and silica nanoparticles are added to the hydrogel’s formulation to increase the mechanical integrity of the actuators.

Our group has surveyed this idea. The same starting materials used to develop electroactive hydrogel-based actuators, which are able to operate outside water, were used to build a pneumatic actuator with self-healing ability and proprioception. The hydrogel not only forms the autonomous self-healable structure of the actuator, but also serves as curvature sensor thanks to its ionic conductivity.^25^

### Tendon-Driven Actuators

This technique is based on an inextensible tendon positioned along the soft actuator. When the tendon is pulled, it generates a motion (usually bending but also twisting and contraction). This method is very used in continuum soft robots made with elastomers, as it results in systems with proper speed/strength responses. As with fluidics, the use of hydrogels to fabricate the actuator could benefit from the smart properties of the material. Our group has partially explored this approach,^22^ developing smart fingertips for a *hard* commercial tendon-driven Barrett-Hand. These fingertips were made of a hydrogel with the ability to change its stiffness. The same approach could potentially be used for a tendon-driven *soft* hand, allowing the hand to change its stiffness.

In conclusion, while there are many stimulus-responsive hydrogel-based demonstrators with potential applicability, nowadays pneumatic and tendon-driven actuators prevail in practical implementations due to their rapid responses. However, pneumatic and tendon-driven actuators require an external source (compressor or other actuators), which complicates and increases the size of the systems. Precisely, the search for compactness and simplicity is what continues to encourage research into improving the smart properties of hydrogels that allow their use in real-world applications.

## Hydrogel-Based Soft Sensors

Soft sensors leverage material properties such as resistive, piezoresistive, capacitive, and optical characteristics.^26^ As with actuators, hydrogel sensors are also influenced by the medium. However, unlike actuators, most applications are found outside of water. For this reason, the authors are seeking a way to prevent water loss from hydrogels. For example, Wang et al. reduced the water loss of a resistive sensor by sandwiching it with a commercial elastomer.^27^ In the following, we present works that explore those characteristics in hydrogels to produce soft-sensors. Moreover, hydrogels provide other characteristics, such as self-healing, biocompatibility, or even a transparent aqueous appearance, which make them a very interesting alternative to soft sensors made of other materials.

### Resistive Strain Sensors

**Figure 6:**
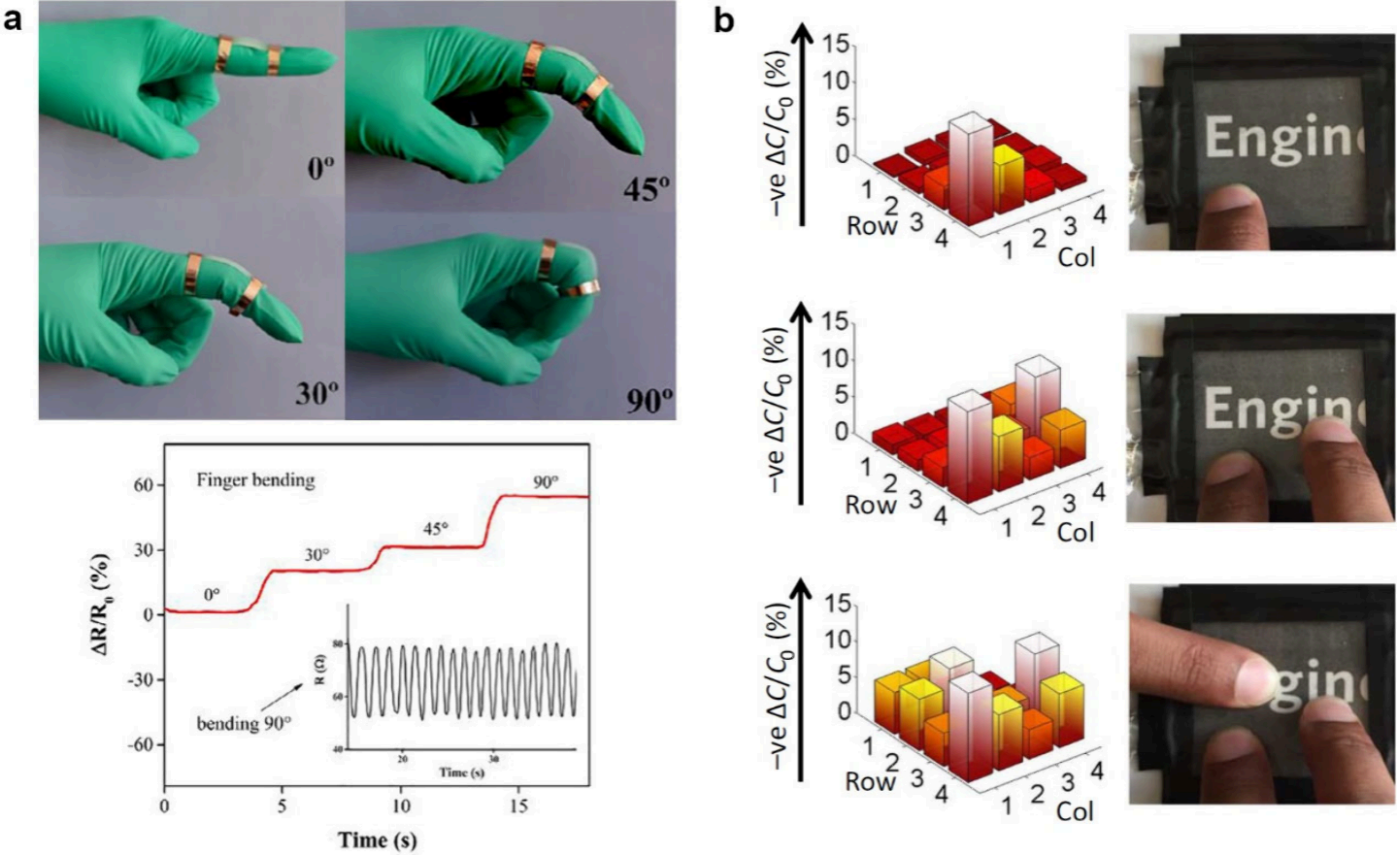
(a) Resistive strain sensor based on an ionic conductive hydrogel was used to measure joint angles. When the finger bends, the hydrogel-based sensor is deformed and its resistance changes, allowing the measurement of the bending angle. Reprinted with permission from ref (33). Copyright 2018 Elsevier. (b) Transparent tactile capacitive sensor based on ionic hydrogels. When there is a contact on the sensor surface, the capacitance changes in the contact zone because of the deformation. Reprinted with permission from ref (35). Copyright 2017 The American Association for the Advancement of Science.

The use of hydrogels in resistive strain sensors has grown a lot in recent years owing to the ease of obtaining a conductive stretchable hydrogel.^28^ Hydrogel-based resistive sensors can be obtained by ionic networks (cationic or anionic)^29^ or conductive polymers,^30^ such as polypyrrole, but also the addition of nanomaterials, such as carbon nanomaterials^31^ or metal nanoparticles,^32^ can provide conductivity to an electrically neutral hydrogel. Several applications can be found for resistive strain sensors: pressure and tactile sensors or joint angle sensors are the most common ones,^27,30,33^ which are usable in manipulator robots, smart skins, or robotic systems intended for motion rehabilitation in humans (Figure 6a). It is the case of the proprioceptive sensor integrated in our pneumatic soft actuator,^25^ which provides the curvature of the actuator, allowing its automatic control. Some works combine conductivity with other stimuli-responses to create multipurpose sensors, like the sensor proposed by Cheng et al.^34^ Thanks to the conductivity and optical transmission of the hydrogel used, the sensor can provide information about different mechanical strains (stretching and twisting) and ambient temperature by using neural networks to analyze all the sensed data.

### Capacitive Sensors

The most common approach to develop capacitive sensors with hydrogels consists of using two conductive hydrogels sandwiched on a dielectric soft layer to form a capacitor with variable capacitance. This kind of sensor has the same applicability as resistive strain sensors. A successful example of a hydrogel-based capacitive sensor is presented in the work of Sarwar et al.,^35^ useful to detect stretching, bending and multitouching (Figure 6b). In this regard, as in the case of resistive sensors, nanomaterials can be used to enhance the conductivity of the hydrogel-based electrodes.^36^ Another approach to develop hydrogel-based capacitive sensors consists of using the hydrogel in the dielectric layer, like the sensor of Wu et al.,^37^ in which a hybrid hydrogel with graphene oxide wrapped by an insulated ultrathin polyethylene film plays the role of the dielectric. In this work, the addition of the 2D material enhances the mechanical properties of the hydrogel and increases the sensor sensibility.

### Optical Sensors

Hydrogels exhibit significant potential as a biocompatible medium for guiding light, offering an alternative to traditional materials, such as glass and inorganic plastics. This feature makes them useful for the manufacturing of optical sensors in a multitude of biosensing and environment sensing applications.^38^ Regarding their use in soft robotics, optical sensors based on hydrogel fibers have the potential to be used as strain sensors to measure curvature or other motions. However, their delicacy can be a handicap for the development of certain applications, such as wearable devices (e.g., rehab exoskeletons). That is why some authors propose methods to improve the strength of the hydrogel, such as the work of Guo et al., where highly stretchable and tough optical fibers made of alginate-polyacrylamide hydrogels are used as strain sensors.^39^

In general, hydrogel-based soft sensors constitute a solid alternative in the field, as some of the main drawbacks can be effectively addressed (e.g., coating to reduce the loss of water or improving their strength through different formulations). Furthermore, the multifunctionality of hydrogels and the additional features they can offer (e.g., self-healing) make them appealing over other materials.

## Manufacturing Possibilities

Although molding is still a frequent manufacturing option, whether using one type of polymerization or another (e.g., heat- or light-activated), the current trend is heading toward 3D printing to prepare hydrogels. This shift is motivated by the possibility of producing complex and intricate structures, which are crucial in soft robotic designs. Nevertheless, not all of the 3D printing methodologies can be implemented for hydrogels, as some conditions can lead to material decomposition. For that reason, the most commonly used techniques are based in extrusion, laser, and inkjet printing. Extrusion-based techniques can be divided into melting-based processes, such as fused deposition modeling and dissolution-based processes, while stereolithography and digital light printing are examples of laser-based printing methodologies. Different reviews cover these techniques.^40^

Among all the 3D printing possibilities, the most versatile for the generation of smart materials are those that allow working with a precursor monomer solution. One example is direct ink writing (DIW), which is based on liquid ink that flows through a nozzle and is solidified by applying a thermal or photocuring treatment. This technique allows multimaterial printing in a simple way, just by adding more nozzles or adjusting the ink composition on the fly,^41^ but the rheological properties of the ink are crucial to obtain shapes with good definition, which restricts the use of many formulations. Based on our experience, the challenge here resides in the design of precursor inks that have the desired properties and also allow for proper 3D printing. For example, in one of our works, we tried to print the starting solution used to form a hydrogel with self-healing capabilities, but the results were unsuccessful due to the low viscosity of the solution, which caused bad layer stacking and low resolution. Reformulation of the ink by including a thickening agent provided a printable solution with good resolution,^16^ but the resulting hydrogel did not exhibit self-healing capabilities.

**Figure 7:**
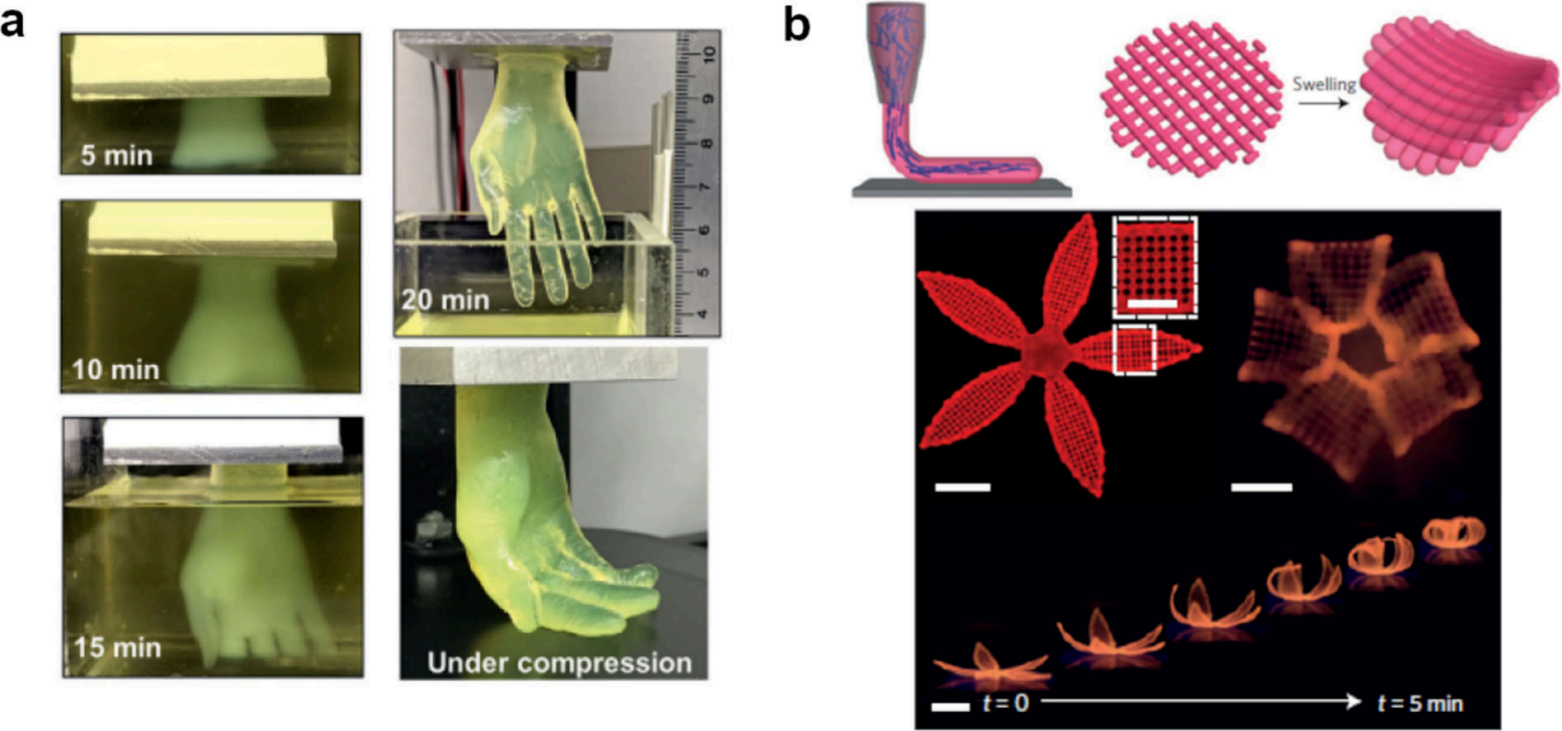
(a) Fast 3D printing of a human hand replica made of hydrogel. The printing technique used is stereolithography. The details of the hand are very precise for such a fast printing operation (only 20 min). Reprinted with permission from ref (42). Copyright 2021 2021 Wiley-VCH GmbH. (b) Example of 4D printing with hydrogels. Thanks to the anisotropy given by the fibrils, which are oriented after flowing through the nozzle, the hydrogel exhibits directional swelling. This allows the printing of parts with different smart movements along time. Scale bar: 5 mm. Reprinted with permission from ref (43). Copyright 2016 Springer Nature.

These rheological drawbacks are not that important in stereolithography (SLA), which is, in fact, the most common methodology for printing hydrogels. In this case, the solution is contained in a vat and does not need to flow through a nozzle, which widens the range of liquid materials that can be used. A platform enters the vat, and UV light beams or a masked UV image (technique known as masked stereolithography, MSLA) polymerizes the solution over the platform. In digital light processing, the photopolymerizable hydrogel precursor is cured via a digital light projection pattern. The problem here is that multimaterial printing is not as straightforward as in DIW, but there exist cases with automatic exchangeable vats for this purpose.^24^ One of the most outstanding results about SLA printing with hydrogels is the one exposed by Anandakrishnan et al.,^42^ in which the authors have achieved fast printing of hydrogels with great microscale details and a replication of a normal-sized human hand in only 20 min (Figure 7a).

Finally, if the printed material has smart features, we can talk about 4D printing, which is the term used to refer to the printed parts that are able to change shape in response to an external stimulus. Hydrogels have been extensively used in this regard. For instance, the work of Gladman et al. shows a hybrid hydrogel with localized swelling thanks to the nanofibrils’ anisotropy achieved during the printing operation (Figure 7b).^43^

## Conclusions and Outlook

Despite the promising start of electroactive hydrogels in the 90s and their early stagnation, hydrogels have once again become part of the soft robotics materials palette. Even so, their presence in soft robotic actuators is not significant in comparison to other materials. So far, pneumatic and tendon-driven actuators are still the main option in the field, and although smart hydrogels can provide additional capabilities to these systems, smart properties are not always required in today’s prototypes.

**Figure 8:**
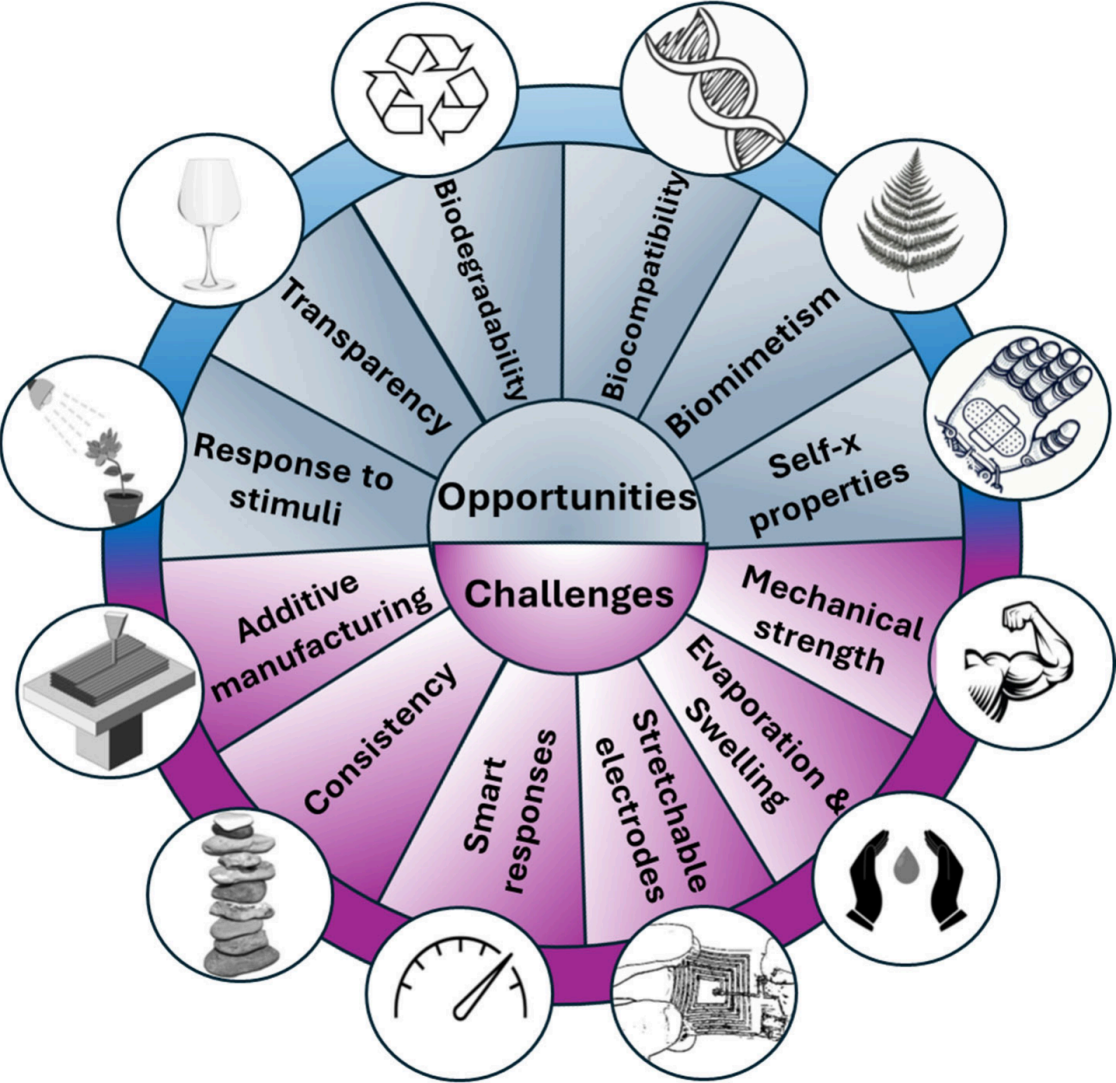
Challenges and opportunities of hydrogels in soft robotics.

Nonetheless, in search of more compact, integral, and untethered soft robots, elements such as air compressors or motors to pull tendons should be removed. It is in this path that hydrogels must play an important role in the upcoming years. The smart features that they exhibit, the additional properties they offer, such as the self-x properties (self-healing, self-sensing, etc.) or the biomimetism, are perfectly suited to the bioinspiration trend, and the huge list of potential existing demonstrators makes these materials an appealing option to evolve in the soft robotics field, despite still being in a low level of practical applicability (refer to Figure 8 for a schematic summary of the challenges and opportunities related to hydrogels).

The great leap of hydrogels in soft robotics must be achieved through an improvement in smart responses and, more importantly, mechanical strength and consistency. Current hydrogel-based actuators whose working principle is based on smart properties do not exhibit enough force for many applications, so, as commented on in the text, the stiffness modulation to achieve actuators with enough strength is a crucial factor. The chemical formulations, the mechanisms to actively regulate the stiffness, and the addition of nanomaterials play a key role in the future of smart actuators based on hydrogels.^44^ However, there is also another way to approach this fact. Perhaps we cannot expect these materials to be used to build the kind of classic robot that everyone has in mind. We may have to be more creative and think of capabilities that are not possible with the rigid materials used so far, robots that perform functions that we have not yet imagined.

On the other hand, hydrogels should overcome the evaporation problem. When working in air conditions, hydrogels exchange water with the environment. Depending on the ambient humidity, their swelling can be different (drier in a dry environment and more swollen in a humid environment), which entails changes in their behavior. This situation, which in some applications could serve as a humidity sensor, is undesirable in other cases. Solutions to keep the swelling constant can be found at the physical level, like coating the hydrogel, or at the material level, through the hydrogel’s formulation or adding nanomaterials that prevent the evaporation.^15,17^

The solution to these issues must be accompanied by a series of improvements in the field of soft robotics that affect not only hydrogels. An example is more refined 3D printing techniques to produce complex shapes with good definition and including multiple materials,^40^ or the development of truly stretchable electrodes with great conductivity, which is something that has been studied for years without getting an outstanding solution that works for all applications. Stretchable electrodes with great conductivity would be of great help in the development of electroactive hydrogel-based actuators as well as improve the efficiency of hydrogel-based sensors and simplify its instrumentation.

Beyond the classical robotics applications, such as manipulator arms or grippers, hydrogels must be considered for many robotics-related applications thanks to the properties they can offer. For instance, biocompatibility and biomimetism (i.e., the resemblance to biological tissues) make them ideal prospects for prosthetics or wearables. That is the real advantage of hydrogels over other materials: the vast variety of features that one material can have all-in-one. And that is the reason why they are utilized in different fields beyond robotics, like medicine (drug delivery, cell culture, tissue engineering), agriculture, or body care and hygienics (diapers, contact lenses, etc.), among others. This versatility favors their insertion not only in known issues but also in future unknown problems.

Another advantage of hydrogels is that they can be biodegradable, promoting environmental sustainability. Furthermore, some hydrogels, as many other polymeric materials, can be decomposed on purpose to reutilize the raw material to generate other hydrogel pieces, reducing material wastes and favoring the circular economy.^45^

All in all, considering the whole view, hydrogels are ideal candidates to take over soft robotics in the upcoming years due to the large list of properties they can exhibit (e.g., response to stimuli, self-healing, transparency, biocompatibility, biomimetism, etc.), which are aligned with bioinspiration in the search for a living organism-like robot. Their future in the field is guaranteed.

## Abbreviations

pNIPAM: poly(*N*-isopropylacrylamide)
pAAM: polyacrilamyde
DIW: direct ink writing
SLA: stereolithography
MSLA: masked stereolithography
